# Mass spectrometry analysis of the variants of histone H3 and H4 of soybean and their post-translational modifications

**DOI:** 10.1186/1471-2229-9-98

**Published:** 2009-07-31

**Authors:** Tao Wu, Tiezheng Yuan, Sau-Na Tsai, Chunmei Wang, Sai-Ming Sun, Hon-Ming Lam, Sai-Ming Ngai

**Affiliations:** 1 Department of Biology and State (China) Key Laboratory of Agrobiotechnology, The Chinese University of Hong Kong, Hong Kong, PR China; 2 Feed Research Institute, Chinese Academy of Agricultural Sciences, Beijing, PR China

## Abstract

**Background:**

Histone modifications and histone variants are of importance in many biological processes. To understand the biological functions of the global dynamics of histone modifications and histone variants in higher plants, we elucidated the variants and post-translational modifications of histones in soybean, a legume plant with a much bigger genome than that of *Arabidopsis thaliana*.

**Results:**

In soybean leaves, mono-, di- and tri-methylation at Lysine 4, Lysine 27 and Lysine 36, and acetylation at Lysine 14, 18 and 23 were detected in HISTONE H3. Lysine 27 was prone to being mono-methylated, while tri-methylation was predominant at Lysine 36. We also observed that Lysine 27 methylation and Lysine 36 methylation usually excluded each other in HISTONE H3. Although methylation at HISTONE H3 Lysine 79 was not reported in *A. thaliana*, mono- and di-methylated HISTONE H3 Lysine 79 were detected in soybean. Besides, acetylation at Lysine 8 and 12 of HISTONE H4 in soybean were identified. Using a combination of mass spectrometry and nano-liquid chromatography, two variants of HISTONE H3 were detected and their modifications were determined. They were different at positions of A^31^F^41^S^87^S^90 ^(HISTONE variant H3.1) and T^31^Y^41^H^87^L^90 ^(HISTONE variant H3.2), respectively. The methylation patterns in these two HISTONE H3 variants also exhibited differences. Lysine 4 and Lysine 36 methylation were only detected in HISTONE H3.2, suggesting that HISTONE variant H3.2 might be associated with actively transcribing genes. In addition, two variants of histone H4 (H4.1 and H4.2) were also detected, which were missing in other organisms. In the histone variant H4.1 and H4.2, the amino acid 60 was isoleucine and valine, respectively.

**Conclusion:**

This work revealed several distinct variants of soybean histone and their modifications that were different from *A. thaliana*, thus providing important biological information toward further understanding of the histone modifications and their functional significance in higher plants.

## Background

Histone modifications and histone variants play critical roles in regulating gene expression, modulating the cell cycle, and are responsible for maintaining genome stability [1-3]. The fundamental structural unit of chromatin in eukaryotic cells is the nucleosome, that consists of 146 base pairs (bp) of DNA wrapped around a histone octamer, each of which is formed by two copies of H2A, H2B, H3 and H4 [4]. An additional histone, H1 links these nucleosomes together along the chromatin chain. In general, the N terminus of histone H3 and H4, and N and C terminus of H2A and H2B are prone to being covalently modified by many enzymes, such as HMT (histone methyltransferase) and HAT (histone acetyltransferase). These modifications include methylation, acetylation, phosphorylation, ubiquitination, glycosylation, ADP ribosylation, carbonylation, sumoylation and biotinylation. Most of these modifications are dynamic and can be reversed by other enzymes, such as histone demethylase and HDAC (histone deacetylase). Using techniques such as Western blotting and mass spectrometry, increasing number of histone modification sites have been identified in mouse, yeast, *Drosophila melanogaster*, *Tetrahymena thermophila *and *A. thaliana *[1-3]. Mass spectrometry (MS) allows us not only to deduce the amino acid sequence of a peptide, but also to identify the exact sites and type of modifications in the peptide via the modified peptide mass shifts.

Epigenetic studies of chromatin in model organisms have provided insights into the modifications of histones, ranging from the identification of several enzymes and related effectors associated with histone modifications to their biological functions in cell development [5,6]. It is currently proposed that histone modifications play vital roles in many fundamental biological processes by rearranging the structure and composition of chromatin. In eukaryotes, such chromatin re-structuring events can help partition the genome into distinct domains such as euchromatin and heterochromatin and result in DNA transcription, DNA repair and DNA replication [7,8]. Nonetheless, some histone modifications may also participate in chromosome condensation, indicating their importantce in the cell cycle and cell mitosis [9,10]. Interestingly, corresponding to their different functions, different histone modifications have different distribution patterns along the chromatin. For example, acetylated histones and methylated histone H3 Lysine 4 mainly locate at the actively transcribing genes [11,12], while histone H3 Lysine 9 methylation is a marker of heterochromatin [13-16].

Although histone modifications and their functions are well studied in yeast and mammals [1-3], similar studies in plants are just at its infancy stage. Recently, the variants of histone H2A, H2B, H3 and H4 and their modifications in *A. thaliana *have been identified using mass spectrometry [3,17,18]. These studies reveal modifications at sites that are unique to plant [17]. The genomic distribution patterns of several histone posttranslational modifications (histone H3 Lysine 4 di-methylation, histone H3 Lysine 9 di-/tri-methylation, and histone H3 Lysine 27 tri-methylation) in *A. thaliana *have been determined by microarray combined with chromatin immunoprecipitation (ChIP-chip), and those distribution patterns are consistent with their functions [19].

Posttranslational modifications (PTMs) can regulate the plant's responses to internal and external signals, such as cell differentiation, development, light, temperature, and other abiotic and biotic stresses [20]. For example, methylation and acetylation of histone H3 regulate the expression of *FRIGIDA (FRI), FLOWERING LOCUS C (FLC) *and other vernalization related genes to ensure flowering at proper time in *A. thaliana *[21-25]. Studies have shown that histone modifications may also be involved in plant responses to abiotic stresses, such as salinity stress and drought stress [26]. In addition, phosphorylation of histone H3 is involved in chromosome condensation and sister chromatid cohesion [27]. Histone acetylation can also affect cellular pattern in Arabidopsis root epidermis by regulating the expression of cellular patterning genes [28]. However, the PTMs of histone in other plant species are still elusive, including several important crops, like soybean, rice and wheat. Investigation on histone epigenetics in other higher plant systems will contribute to deciphering of the "histone code" hypothesis [29].

Soybean is an important economic crop with a diploidized tetraploid genome (~950 Mb) which is much larger than that of *A. thaliana *(125 Mb) [30]. Here, we report the first identification of the variants of soybean histone H3 and H4 and their PTMs using matrix-assisted laser desorption/ionization-time-of-flight mass spectrometry (MALDI-TOF MS), in combination with nano-liquid chromatography (nano-LC). Our investigations reveal some important features of histone modifications in soybean, including acetylation at histone H3 Lysine 14, Lysine 18, Lysine 23; and histone H4 Lysine 8 and Lysine 12; methylations at histone H3 Lysine 4, Lysine 27 and Lysine 36. Surprisingly, histone H3 Lysine 79 is also methylated in soybean, which is not reported in *A. thaliana *[17]. In addition, variants of histone H3 (H3.1 and H3.2) and histone H4 (H4.1 and H4.2) are also identified and different modifications of the two variants of histone H3 are also studied.

## Results

### Isolation and identification of core histones of soybean

**Figure 1:**
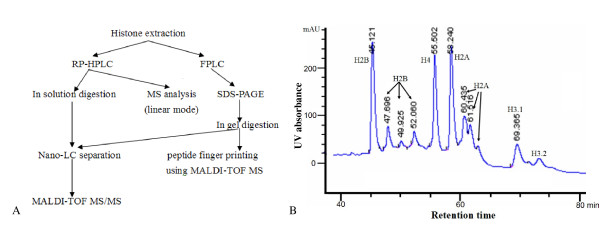
**Isolation and purification of soybean core histone from leaves with RP-HPLC and FPLC**. A: Strategies used in this experiment. B: Spectrum of histone isolation with RP-HPLC. The core histones were extracted in acid and separated by RP-HPLC. They were eluted in the sequence of histone H2B, H4, H2A and H3, while histone H1 was not isolated. Several variants of histone H2B, H2A and H3 were separated and their retention times were labeled on the top of their corresponding peaks.

Using reversed phase high-performance liquid chromatography (RP-HPLC), core histones of soybean were separated and eluted in the order of H2B, H4, H2A and H3 between 38–55% of buffer B, and collected according to the UV signal (210 nm) (Figure 1A). MALDI-TOF MS (linear mode) was employed to monitor the isolated histones in the collected fractions and the calculated mass of histone H4, H3, H2A and H2B were approximately 11.3, 15.2, 15.3 and 16.1 kDa, respectively. According to the results of the RP-HPLC analysis (Figure 1B), several variants of H2B and H2A were detected. Triton-urea-acetic acid (TUA) gel indicated that at least 5 variants of histone H2B and 4 variants of histone H2A were present in soybean (data not shown). By extending the slope of gradient of buffer B from 35% to 65% ACN in 100 min, two variants of histone H3, H3.1 and H3.2, were also separated (Figure 1B).

Core histones were also isolated by fast protein liquid chromatography(FPLC) and the individual histone protein was then separated via SDS-PAGE (Figure 1A). Protein bands containing the corresponding core histones were excised and followed by endoproteinase in-gel digestion. Each histone protein band was divided into two portions and subjected to trypsin or Lys-C digestion respectively before MS analysis. MS analysis covered most of the amino acid sequence of histone H3, which consists of 135 amino acid residues. Most of the 102 amino acid residues in soybean histone H4 were also identified using MS analysis.

### Histone modifications of soybean histone H3 and its variants

**Figure 2:**
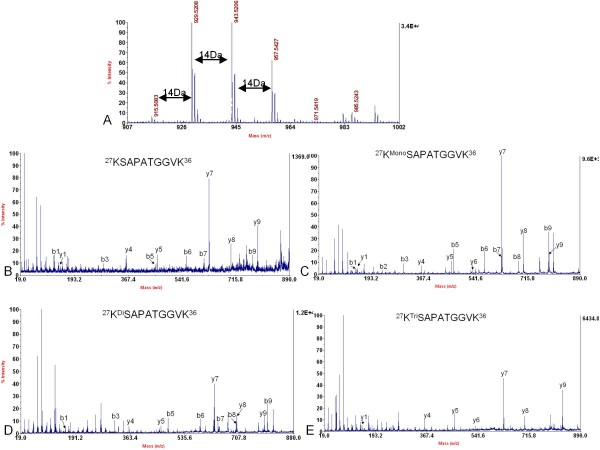
**Determination of histone variant H3.1 and identification of methylation at Lysine 27 of histone variant H3.1**. A. MALDI-TOF mass spectrum showing non- (*m/z *915.52), mono- (*m/z *929.53), di- (*m/z *943.53) and tri- (*m/z *957.55) methylation at Lysine 27 in the peptide ^27^KSAPATGGVK^36 ^of histone H3.1. B, C, D and E. MS/MS spectrum of the peptide precursor ions at *m/z *915.52, 929.53, 943.53 and 957.55 determining non-, mono-, di- and tri-methylation at Lysine 27 in the peptide of ^27^KSAPATGGVK^36 ^of histone H3.1, respectively. These results clearly showed that the amino acid sequence of this peptide was KSAPATGGVK and only Lysine 27 was methylated, but not Lysine 36.

**Figure 3:**
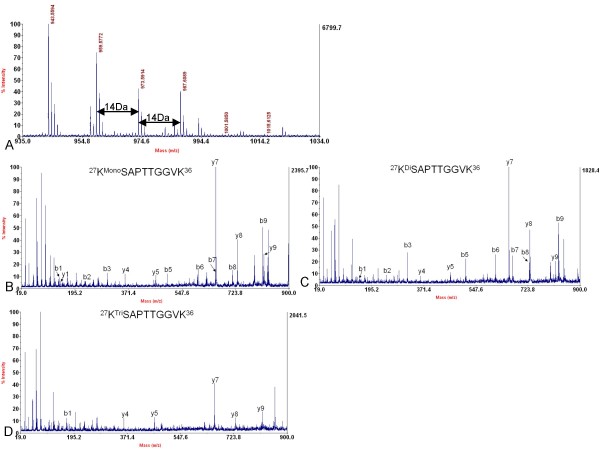
**Determination of histone variant H3.2 and identification of methylation at Lysine 27 of histone variant H3.2**. A. MALDI-TOF mass spectrum showing mono- (*m/z *959.58), di- (*m/z *973.59) and tri- (*m/z *987.61) methylation at Lysine 27 in the peptide ^27^KSAPTTGGVK^36 ^of histone H3.2, but without non-methylation (about *m/z *945) at this site. B, C and D. MS/MS spectrum of the peptide precursor ions at *m/z *959.58, 973.59 and 987.61 respectively determining mono-, di- and tri-methylation at Lysine 27 in the peptide of ^27^KSAPTTGGVK^36 ^of histone H3.2. B, C, and D indicated that the amino acid sequence of this peptide was KSAPTTGGVK and only Lysine 27 was methylated, but not Lysine 36.

**Figure 4:**
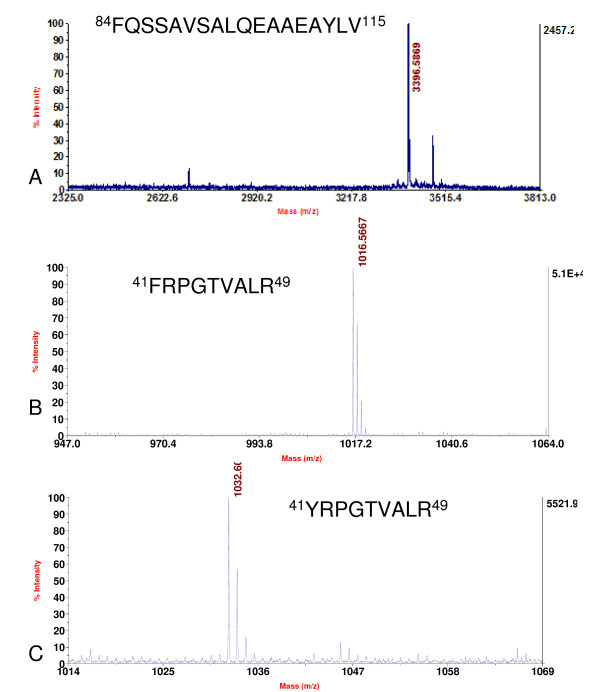
**Confirmation of two variants of histone H3 of soybean**. A and B. MALDI-TOF mass spectrum showing the peptide precursor ions at *m/z *3396.60 and 1016.57 corresponding to the peptide ^84^FQSSAVSALQEAAEAYLV^115 ^and ^41^FRPGTVALR^49 ^of histone variant H3.1 respectively. C. MALDI-TOF mass spectrum showing the peptide precursor ion at *m/z *1032.60 corresponding to the peptide ^41^YRPGTVALR^49 ^of histone variant H3.2.

**Figure 5:**
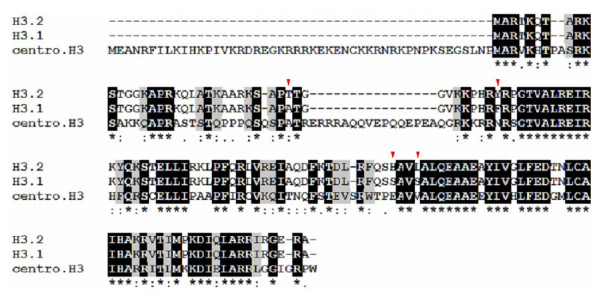
**Protein sequence alignment of the three variants of histone H3 in soybean**. The cetromere specific histone H3 (centro. H3) was very different from the other two histone variants H3.1 and H3.2, while H3.1 and H3.2 were different from each other in only 4 amino acids, A^31^F^41^S^87^S^90 ^in H3.1 and T^31^Y^41^H^87^L^90 ^in H3.2, which were indicated by red triangle in the figure. Sequences were downloaded from soybean genome database . Accession numbers were as follows: Glyma11g37960.1 for histone H3.2; Glyma11g13940.1 for histone H3.1; Glyma07g06310.1 for centro.H3.

Two variants of histone H3 were determined in soybean. Although the amino acid sequences of the two variants of *D. melanogaster *histone H3 were very similar and with only four amino acid differences, they could be separated by extending the slope of gradient of buffer B during RP-HPLC separation [31]. Similar methods were adopted to isolate soybean histone H3 variants (Figure 1B). Two consecutive peaks were eluted between 46.2% – 47.2% of buffer B. These two peaks were collected, digested by trypsin and analyzed by nano-LC/MS/MS separately. In the mass spectrum of the first peak, the histone peptide with the mass of 929.53 containing ^27^KSAPA^31^TGGVK^36 ^was detected (Figure 2). In the mass spectrum of the second peak, another histone peptide with the mass of 959.58, corresponding to ^27^KSAPT^31^TGGVK^36 ^was identified (Figure 3). These two histone peptides were different in the amino acid residue 31, so the first and second peaks were designated histone H3.1 and H3.2, respectively. We further analyzed the variants of histone H3 using the information from soybean genome database . Data from soybean genome showed that these two histone H3 variants in soybean differed in four amino acids at the position of amino acid 31, 41, 87 and 90. They were A^31^F^41^S^87^S^90 ^and T^31^Y^41^H^87^L^90 ^in histone H3.1 and H3.2, respectively. Three more peptides from our MS analysis further confirmed this conclusion: peptide precursor ion at *m/z *3396.60 containing ^84^FQSS^87^AVS^90^ALQEAAEAYLV^115 ^and peptide precursor ion at *m/z *1016.57 containing ^41^FRPGTVALR^49 ^in the mass spectrum of histone H3.1, peptide precursor ion at *m/z *1032.60 corresponding to ^41^YRPGTVALR^49 ^in the mass spectrum of histone H3.2 (Figure 4). In the soybean genome, we also found another histone H3 variant, centromere specific histone H3, which differed greatly in amino acid sequence from the other two variants (Figure 5).

**Figure 6:**
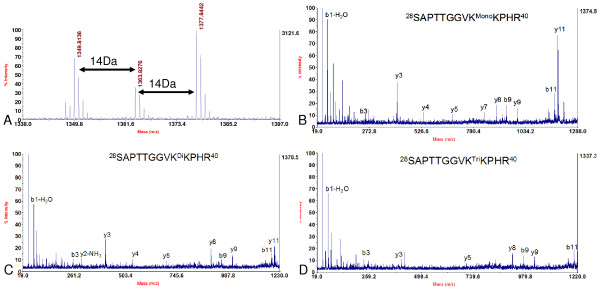
**Identification of methylation of Lysine 36 of histone variant H3.2**. A. MALDI-TOF mass spectrum showing mono- (*m/z *1349.81), di- (*m/z *1363.83) and tri- (*m/z *1377.84) methylation at Lysine 36 of histone H3.2. B, C and D. MS/MS spectrum of the peptide precursor ions at *m/z *1349.81, 1363.83 and 1377.84 which determined mono-, di- and tri-methylation at Lysine 36 of histone H3.2, respectively.

**Figure 7:**
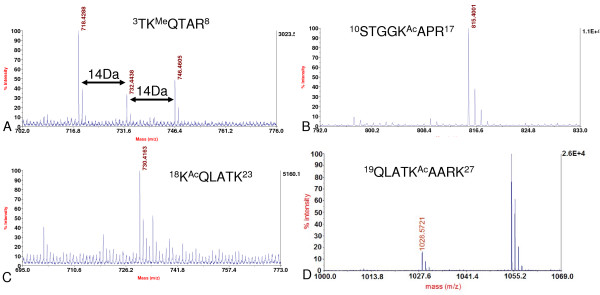
**Identification of modification sites of histone H3**. A. MALDI-TOF mass spectrum showing mono- (*m/z *718.43), di- (*m/z *732.44), tri- (*m/z *746.46) methylation at Lysine 4 of histone H3. B. MALDI-TOF mass spectrum showing acetylation (*m/z *815.40) at Lysine 14 of histone H3. C. MALDI-TOF mass spectrum showing acetylation (*m/z *730.42) at Lysine 18 of histone H3. D. MALDI-TOF mass spectrum showing acetylation (*m/z *1028.57) at Lysine 23 of histone H3. Me: methylation; Ac: acetylation.

**Figure 8:**
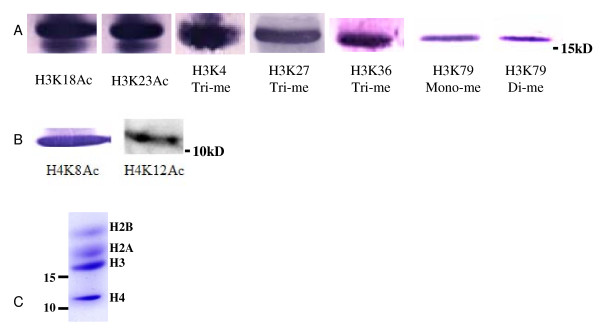
**Identification of histone modifications in histone H3 and H4 by Western Blotting**. Ten μg soybean core histone mixtures were separated in 15% SDS-PAGE gel, and transferred to a PVDF membrane (one μg samples were used when antibodies that recognized H3K18Ac and H3K23Ac were used). A. Western blotting showed the presence of H3K18Ac, H3K23Ac, H3K4Tri-me, H3K27Tri-me, H3K36Tri-me, H3K79Mono-me and H3K79Di-me in histone H3. B. Western blotting showed the presence of H4K8Ac and H4K12Ac in histone H4. C. Coomassie stained SDS-PAGE gel showed the soybean core histone H2A, H2B, H3 and H4. Specific antibodies used were marked under their corresponding figure. Ac: acetylation; Me: methylation.

Next, the modifications of histone H3 were investigated. Modifications of histone H3 were complicated due to its high abundance of both Lysine and arginine in its primary amino acid sequence (Table 1). From the MS analysis, mono-, di- and tri-methylation of Lysine 27 were detected in both histone H3 variants; with mono-methylation as the predominant modification (Figure 2 and 3). In the trypsin digestion, peptide precursor ions with the mass of *m/z *959.58, 973.59 and 987.61 represented the mono-, di-, and tri-methylated peptides ^27^KSAPTTGGVK^36 ^of histone variant H3.2 respectively (Figure 3). Although such peptide contained two potential methylation sites (Lysine 27 and Lysine 36), *de novo *sequencing clearly indicated that methylation were mainly located at Lysine 27 (Figure 3). Methylated Lysine 36 was determined by other peptides whose mass were *m/z *1349.81, 1363.83 and 1377.84 containing ^28^SAPTTGGVKKPHR^40 ^of histone variant H3.2. *De novo *sequencing showed that it could also be mono-, di- and tri-methylated (Figure 6). More interestingly, most of histone H3 Lysine 36 methylation did not appear in those peptides which contained histone H3 Lysine 27 methylation, since only two very small peaks whose mass were *m/z *1001.59 and 1015.61 were detected in the MS spectrum (Figure 3A), which may be corresponding to the peptides containing methylation at both Lysine 27 and Lysine 36. In addition, no peptide that contained both tri-methylated Lysine 27 and Lysine 36 was identified because of the absence of peptide precursor ion at *m/z *1029 in Figure 3A. Similar results were also obtained in histone variant H3.1 (Figure 2). Other PTMs were also observed in the peptides of histone H3. Peptide ^3^TKQTAR^8 ^containing mono-, di- and tri-methylated histone H3 Lysine 4, of which mass were *m/z *718.43, 732.44 and 746.46 respectively, were detected (Figure 7A). Of these three modifications, histone H3 Lysine 4 mono-methylation was the dominant one, and this result was similar to that in *A. thaliana *[17]. Lysine acetylation in soybean histone H3 was also identified. Peptides ^10^STGGK^14Ac^APR^17 ^at the *m/z *815.40 and ^18^K^Ac^QLATK^23 ^at the *m/z *730.42 containing acetylated Lysine 14 and Lysine 18 respectively were shown in Figure 7B and 7C. Another peptide at the *m/z *1028.57 containing acetylated Lysine 23 was also detected, which was ^19^QLATK^23Ac^AARK^27 ^(Figure 7D). Since the mass shift of acetylation and tri-methylation were very similar (~42 Da), Western blotting with specific antibodies to these acetylation and tri-methylation sites was performed and further confirmed our MS results (Figure 8A).

**Table 1:** Comparison of PTMs of histone H3 in *Glycine max*, *A. thaliana *and mammals

		Modification Sites			Functions
		mammals	*A. thaliana*	*G. max*	
Acetylation	K9	+	+	nd	Transcriptional activation
	K14	+	+	+	Transcriptional activation
	K18	+	+	+	Transcriptional activation
	K23	+	+	+	Transcriptional activation
	K56	nd	+	nd	
Methylation	K4	+	+	+	Transcriptional activation
	K9	+	+	nd	Transcriptional repression
	K27	+	+	+	Transcriptional repression
	K36	+	+	+	Transcriptional activation
	K64	+	nd	nd	
	K79	+	nd	+	Telomere silencing
	K122	+	nd	nd	

nd, not detected; +, modification present.

**Figure 9:**
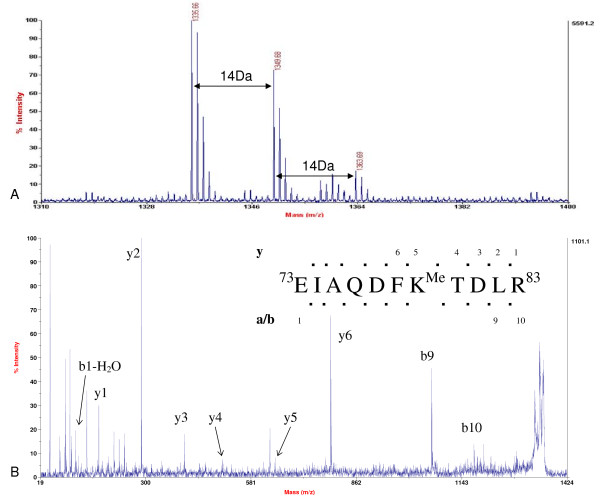
**Identification of methylation at Lysine 79 of histone H3**. A. MALDI-TOF mass spectrum showing non- (*m/z *1335.66), mono- (*m/z *1349.68), and di- (*m/z *1363.69) methylation at Lysine 79 of histone H3. B. MS/MS spectrum of the peptide precursor ion with the mass 1349.68, demonstrating mono-methylation at Lysine 79 in the peptide ^73^EIAQDFK^79^TDLR^83^. However, our data did not indicate that whether histone H3 Lysine 79 methylation was located in certain histone H3 variant.

Methylation of histone H3 Lysine 79 was observed in our studies. Such methylation was frequently found in mammals [32]. Compared with the mass of the peptide at *m/z *1335.66, the mass of the peptides at *m/z *1349.68 and 1363.69 shifted about 14 Da and 28 Da (Figure 9A). This indicated that these peptides might be methylated. Fragmentation of the methylated peptide at *m/z *1349.68 resulted in a MS/MS spectrum containing both complete b-ion series and y-ion series. According to this spectrum (Figure 9B), the amino acid sequence of ^73^EIAQDFK^79Mono^TDLR^83 ^could be assigned to this peptide, which revealed that there was mono-methylation at Lysine 79 in soybean histone H3. Western blotting was performed to confirm this result (Figure 8A). Consequently, the peptide with the mass 1363.69 should contain di-methylated histone H3 Lysine 79. Due to their low abundance, *de novo *sequence was not successful; however, Western blotting supported this prediction (Figure 8A).

The differences of the modification patterns found in these histone H3 variants were obvious. Although most of their acetylation patterns were similar, their methylation patterns exhibited several differences. Almost all of Lysine 27 in histone variant H3.2 were methylated, whereas some histone variant H3.1 were not methylated at Lysine 27. A peptide precursor ion at *m/z *915.49 which contained the unmethylated Lysine 27 was detected in histone H3.1 (Figure 2A) while the peptide containing unmethylated Lysine 27 of histone H3.2 (with a theoretical mass about 945) were absent in Figure 3A. On the other hand, the peptide containing unmethylated Lysine 36 was not detected in both histone H3 variants. While Lysine 36 methylation can be easily detected in histone H3.2 (Figure 6), such methylation was not detected in histone H3.1. Another difference between these two variants was that mono-, di- and tri- methylated Lysine 4 were also only present in histone H3.2 (Figure 7A). Although the modifications of the soybean centromere specific histone H3 were not identified in this study, the amino acid residues at all the acetylated sites and two methylated sites (Lysine 27 and Lysine 79) of histone H3.1 and H3.2 were different in the centromere specific histone H3 (Figure 5), indicating that the centromere specific histone H3 might have distinct histone modification patterns from that of H3.1 and H3.2.

### Histone modifications of soybean histone H4 and its variants

**Figure 10:**
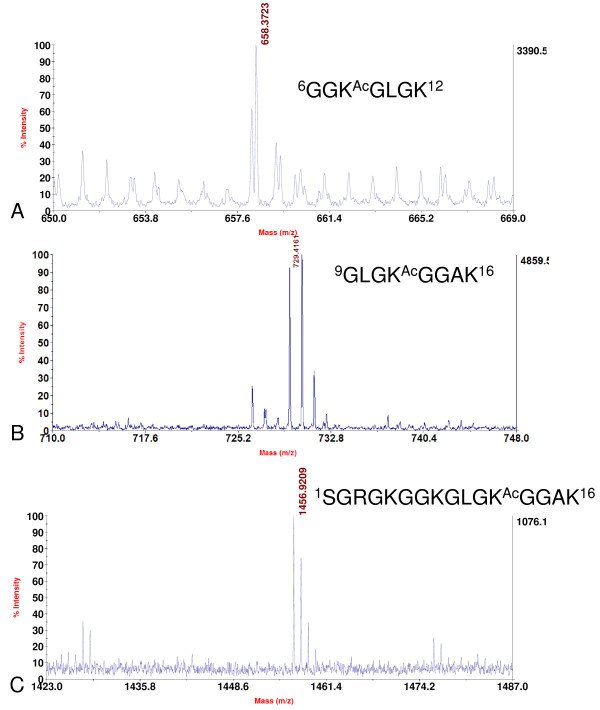
**Identification of acetylation sites in histone H4**. A. MALDI-TOF mass spectrum showing the acetylation (*m/z *658.37) at Lysine 8 of histone H4. B and C. MALDI-TOF mass spectrum showing the acetylation (*m/z *729.42 and 1456.92 respectively) at Lysine 12 of histone H4. Ac: acetylation.

Purified histone H4 was digested separately with either trypsin or Lys-C and the corresponding digested fractions were separated and analyzed by nano-LC combined with MS/MS. Most of the potential PTM sites were examined and compared to other species. Acetylation of histone H4 was observed. As shown in Table 2, Lysine 8 of histone H4 was acetylated in the peptide ^6^GGK^8Ac^GLGK^12 ^with the mass of 658.37 (Figure 10A). Lysine 12 was acetylated in the histone H4 peptide ^9^GLGK^12Ac^GGAK^16 ^with mass at *m/z *729.42 (Figure 10B). None of the two unacetylated or di-acetylated peptide precursor ions was detected. We also detected a peptide precursor ion with mass at *m/z *1456.92, which corresponded to the peptide ^1^SGRGKGGKGLGK^12Ac^GGAK^16 ^(Figure 10C) and further proved that Lysine 12 could be acetylated. Similarly, these acetylation sites were further verified by Western blotting with specific antibodies to histone H4 Lysine 8 acetylation and Lysine 12 acetylation (Figure 8B). However, acetylation of Lysine 5 and 16 were not detected. Our data thus indicated that Lysine 8 and 12 were the main acetylation sites in the N terminus of soybean histone H4 and their acetylation might not happen simultaneously; a result that is differed from those found in histone H4 of *A. thaliana *and mammals [17]. In our MS analysis, we cannot detect histone H4 Lysine 20 modification, whereas the Western blotting results showed that histone H4 Lysine 20 methylation did present in soybean (data not shown).

**Table 2:** Comparison of PTMs of histone H4 in *Glycine max*, *A. thaliana *and mammals

		Modification Sites			Functions
		Mammals	*A. thaliana*	*G. max*	
Acetylation	K5	+	+	nd	Transcriptional repression
	K8	+	+	+	Transcriptional activation
	K12	+	+	+	Transcriptional activation
	K16	+	+	nd	Transcriptional activation
	K20	nd	+	nd	
Methylation	K20	+	nd	+	Heterochromatin silencing

nd, not detected; +, modification present.

**Figure 11:**
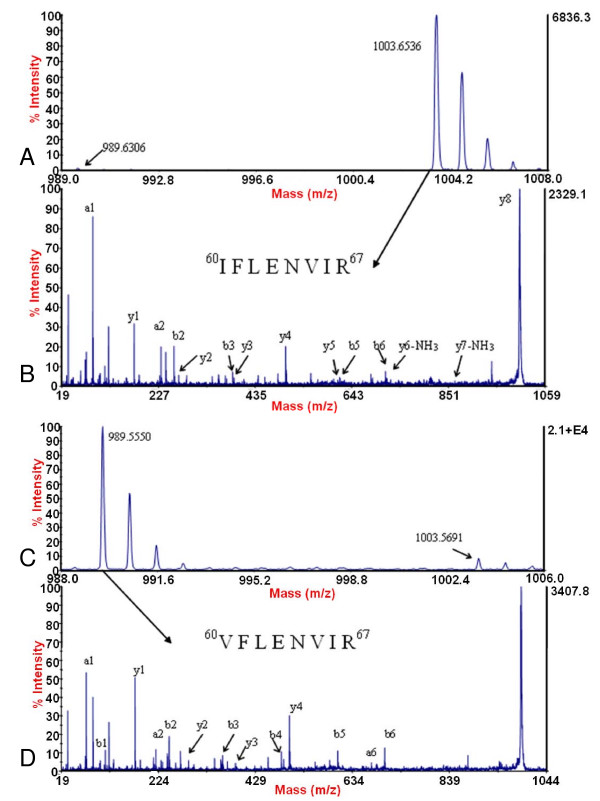
**Identification of the two variants of histone H4**. A. MALDI-TOF mass spectrum showing that the amount of the peptide with calculated mass of *m/z *1003.6 from histone H4.1 was much more than that of the peptide (*m/z *989.6) from histone H4.2 in the peptide mass fingerprinting of trypsin digested histone H4. B. MS/MS spectrum showing peptide (*m/z *1003.6) corresponding to ^60^IFLENVIR^67 ^of histone variant H4.1. C. MALDI-TOF mass spectrum showing the peptide (*m/z *989.6) from histone H4.2 after nano-LC separation. D. MS/MS spectrum showing the peptide (*m/z *989.6) corresponding to ^60^VFLENVIR^67 ^of histone variant H4.2.

Two variants of histone H4 were identified (designated as H4.1 and H4.2), which varied at the amino acid residue I^60 ^of histone H4.1 and V^60 ^of histone H4.2 (Figure 11). The trypsin digested peptides of histone H4 were directly applied to MALDI-TOF/TOF analysis and after peptide mass fingerprinting search, the peptide precursor ion at *m/z *1003.65 was readily detected. Further *de novo *sequencing showed that it contained the amino acid sequence of ^60^IFLENVIR^67^. However, in the nano-LC fractionated histone H4 peptides, another peptide with the amino acid sequence of ^60^VFLENVIR^67 ^with the mass of 989.55 was detected. Although only one peak representing histone H4 was observed in the RP-HPLC spectrum (Figure 1B), it may be due to the high similarity in the hydrophobicity of the two variants so that they can not be separated using such method.

## Discussion

In general, the amino acid sequences of histones in eukaryote are highly conserved and the posttranslational modification (PTM) patterns on specific amino acid residues are also quite similar. Characterization of histone modifications of histones H3 and H4 in soybean showed similarities to that of *A. thaliana *and other organisms. High density acetylations in the N-terminal tails of histone H3 and H4 were detected in both soybean and other organisms [1-3,9]. It is suggested that these acetylations play important roles in the transcriptional regulation of many physiological processes in plants, including cold tolerance, floral development and light responsiveness [33,34].

However, histone modification patterns in different eukaryotes may also have some distinct properties. For example, previous studies indicated that histone H4 Lysine 20 modifications were quite distinct between animal and plant. Histone H4 Lysine 20 methylation is evolutionarily conserved from yeast to mammals and is very critical in DNA repair and genome integrity [35]. However, histone H4 Lysine 20 was acetylated in *A. thaliana *[17]. Our results also showed some differences that exist between soybean and the model dicot *A. thaliana*: mono- and di- methylation of Lysine 79 were detected in soybean but such PTMs were not found in *A. thaliana *[17]. Western blotting results also showed that methylated histone H3 Lysine 79 might not be widely distributed throughout the whole soybean genome, since when equal amount of histone was applied, the signals of histone H3 Lysine 79 methylation were much weaker than that of other modifications of histone H3 (Figure 8A). Studies in yeast and mammals show that histone H3 Lysine 79 is hypermethylated at silenced loci and is important in DNA repair and genome stability [1,36]. Whether this modification is also crucial in maintaining soybean genome integrity requires further investigations.

The patterns of histone H3 Lysine 27 and Lysine 36 methylation were also different between soybean and *A. thaliana*. Previous studies indicate that methylation of Lysine 27 and Lysine 36 carry independent functions: Histone H3 Lysine 27 methylation is mainly involved in gene silencing and heterochromatin formation while methylated histone H3 Lysine 36 is found to be associated with the phosphorylated CTD of Pol II, suggesting a role in gene expression and elongation [37]. In *A. thaliana*, the MADS-box transcription repressor FLOWERING LOCUS C (FLC) is a crucial regulator in controlling flowering time. Histone H3 Lysine 27 methylation usually represses FLC expression while histone H3 Lysine 36 methylation has an opposite effect, suggesting that the modifications at these two sites must be carefully regulated in order to flower properly [23,25,38]. In *A. thaliana*, it was reported about 15% of the peptides from histone variant H3.2 were modified with both histone H3 Lysine 27 di-methylation and Lysine 36 mono-methylation [3]. So it seems that methylated Lysine 27 and Lysine 36 can coexist on the same histone H3 N-terminus in *A. thaliana*. However, our present MS data revealed that most of the methylated Lysine 27 and methylated Lysine 36 were unlikely to coexist on the same histone H3 molecule in soybean. Therefore, we speculate that soybean and *Arabidopsis *may regulate the occurrence of histone H3 Lysine 27 and Lysine 36 methylation by different ways, although so far little about the relationship between histone H3 Lysine 27 and Lysine 36 has been known.

Analysis of the public database of soybean genome revealed that at least 14 variants of H2A and 12 variants of H2B were present in soybean. It may be due to the gene duplications and reshuffling events happened during soybean diploidized tetraploid genome formation, which occurred at about 8–10 million years ago and 40–50 million years ago respectively . However, we have not identified any PTMs of soybean histone H2B and H2A in our studies so far.

Genomic analysis also found 3 variants of histone H3 in soybean: H3.1, H3.2 and centromere specific histone H3, but we could not isolate the centromere specific histone H3. Other studies indicate that the expression of this variant peaks in late S/G2 and it is mainly deposited at functional centromeres [39,40]. It may account for the absence of centromere specific histone H3 in soybean leaves which do not undergo active cell division. The modification patterns of the other two histone H3 variants in soybean were different from those in *A. thaliana*. Only tri-methylation at histone H3 Lysine 36 was found in histone H3.1 of *A. thaliana *[3] while methylated histone H3 Lysine 36 including tri-methylation was absent in soybean histone H3.1 and mono-, di- and tri-methylation of histone H3 Lysine 36 were found in soybean histone H3.2. Besides, histone H3 Lysine 4 methylation was only detected in histone variant H3.2. Histone H3 Lysine 4 methylation is suggested to be associated with euchromatin region and viewed as a marker of transcriptionally active genes [11,12]. In addition, methylated Lysine 36 is also associated with gene transcription [37]. Previous studies suggested that different variants of histone H3 might carry different functions [41,42]. In *D. melanogaster *and *A. thaliana*, the replication-independent histone H3 variants which are usually associated with actively transcribing regions are rich in active modifications, including histone H3 Lysine 4 methylation and acetylations [3,31]. The presence of modifications (methylation at Lysine 4 and Lysine 36 and acetylation) in soybean histone H3.2 suggested that the soybean histone H3.2 might also be related to actively transcribing genes.

Two soybean histone H4 variants were identified in our study, although histone H4 was the most conserved core histone, and no variant of histone H4 was found previously [4]. The significance of these two novel histone H4 variants of soybean awaits further investigations.

Our study expands the map of histone PTMs in higher plant. However, some PTMs identified in other organisms, such as the histone H3 Lysine 9 and histone H4 Lysine 20 modifications were not detected in our MS analysis. Our western blotting results indicated that the above PTMs did present in soybean (data not shown). The sensitivity of our existing MS machine may limit the coverage of our study. Therefore, more sensitive and higher resolution MS machinery is definitely preferred for future consideration. Besides, histone phosphorylation was also not detected because the phospho-histones could decompose when they were extracted by acid [17]. In addition, since individual histone PTMs may vary in different tissues and developmental stages, our mass spectrometry analysis here may not be capable of identifying all modification sites along the amino acid sequence of every histone in soybean.

## Conclusion

We present the first report of histone H3 and H4 variants and their PTMs in the legume plant soybean using nano-LC combined with mass spectrometry, mainly focusing on the acetylation and methylation of histone H3 and H4 and their variants. Significant differences are found in histone modifications between soybean and *A. thaliana*, which show that although the amino acid sequences of histones are conserved in evolution, their modification patterns can be quite different. The modifications in the variants of soybean histone H3 are also different, further proving that histone variants have distinct biological functions which are consistent with their specific modification patterns. Our results present comprehensive information for future studies on understanding the biological functions of histone modifications in soybean, such as regulating the DNA transcription and DNA repair. Further investigations in soybean histone modifications may shed light to better understanding the mechanism of epigenetics in plant, a task that cannot be accomplished by solely investigating *A. thaliana*.

## Methods

### Plant growth conditions

Soybean (*Glycine max *[L.] Merr. Cultivar Union) was germinated in soil under greenhouse conditions. One week later, the plants were transferred and cultured in 1× Hoagland nutrient solution. At the growth stage with 3–4 leaves, the leaves were harvested, frozen immediately in liquid nitrogen and stored at -80°C.

### Nuclei extraction and histone isolation

Soybean tissues were ground into powder in liquid nitrogen, and suspended in nuclei isolation buffer (NIB) containing 20 mM Tris-HCl (pH 7.5), 10 mM KCl, 10 mM MgCl_2_, 6% sucrose, 0.6% Triton X-100, 0.05% β-mercaptoethanol, 1 mM phenylmethylsulfonyl fluoride (PMSF), as described (with some modifications) previously [43]. After being homogenized on ice bath, the tissue was filtered using filter paper (pore size 30 μm). The resulting nuclei fraction was harvested by centrifugation at 4000 *g *for 10 min, and then washed twice with NIB. The white nuclei were re-suspended in 40% guanidine hydrochloride. Core histones were then extracted by adding 8 M HCl to 0.4 M at its final concentration. The extract was then centrifuged at 12000 *g *for 10 min. The supernatant containing core histones were dialyzed against 50 mM acetic acid in ice bath, and dried upon the speed vacuum system (SpeedVac). The dried powder of core histone mixture was stored at -20°C until use.

Core histone proteins were separated using the FPLC Duo-Flow system (Bio-Rad, USA), using the C4 column (4.6 × 250 mm, Alltech). The final separation was performed at a flow rate of 1 ml/min with mobile phase A containing 5% methanol and 0.1% trifluoroacetic acid (TFA) in water and mobile phase B containing 40% methanol, 60% acetonitrile (ACN) and 0.1% TFA in water, using a linear gradient program (40%–70% mobile phase B in 30 min). The eluted fractions were finally SpeedVac dried.

In order to separate the two variants of histone H3, 100 μg of purified core histones dissolved in water were separated by reversed-phase high performance liquid chromatography (RP-HPLC) (Agilent 1100 series) using C4 column (4.6×250 mm; 5 μm) [44]. The running program was: buffer A 10 min; 35% to 65% buffer B in 100 min, then 65% to 100% buffer B in 10 min. Buffer A was 0.1% TFA in water; Buffer B was 0.05% TFA in ACN.

### Histone protein in-gel digestion and nano-liquid chromatography

The purified histone powder was re-dissolved using 1 × SDS-PAGE sample loading buffer and subjected to SDS-PAGE analysis (T = 15%). Corresponding histone bands were excised and cut into small pieces. The gel was de-stained twice using the destaining buffer (50% methanol, 50 mM Na_2_CO_3 _in water), dehydrated using ACN and then dried by SpeedVac for 5 min. The de-stained gel chips were immersed in 10–15 μl endoproteinase (15 ng/μl trypsin (Promega) or 5 ng/μl Lys-c (Roche)) and after overnight digestion at 30°C, the gel was sonicated (135W, 42 KHz) for 10 min to extract the digested peptides. After centrifugation, 0.8 μl aliquots of the supernatants were spotted onto the MALDI sample plate and dried in air, followed by adding 0.5 μl of the matrix solution containing α-yano-4-hydroxycinnamic acid in 50% ACN/0.1% TFA for MS analysis.

The digested gel pieces were also suspended in 100 μl 50% ACN/2.5% TFA, sonicated for 10 min and centrifuged at 12000 *g *for 1 min. The supernatant was transferred into new eppendorf tube and dried by SpeedVac. The dried samples were re-dissolved in 50–100 μl buffer A (2% ACN, 0.05% TFA in water) and separated by Nano-LC which was automatically performed using the C18 microcolumn (PrepMap100 3 μm, 15 cm×75 μm, LC Packings, Dionex) on the nano-LC Packings UltiMate™ systems (UltiMate System SwitchosII, Advanced Microcolum Switching Unit, FAMOSII™ Microautosampler, Probot™ MicroFraction Colletor). The elution of peptides was accomplished adopting a linear gradient from 30% mobile phase buffer A to 90% buffer B (80% ACN, 0.05% TFA in water) in 90 min at a flow rate of 0.3 μl/min. Each fraction was autocollected on the MALDI-TOF sample plate.

### Mass spectrometry

Mass spectrometric analysis was carried out using a MALDI-TOF/TOF tandem mass spectrometer ABI 4700 proteomics analyzer (Applied Biosystems, USA). Mass data acquisitions were piloted by 4000 Series Explorer™ Software v3.0. Linear mode MS were operated over the mass range 5 k-25 k *m/z *for full protein detection. Reflector mode MS survey scan were acquired over the mass range 600–3500 *m/z *in the positive-ion mode and accumulated from 2000 laser shots with acceleration of 20 kV. The MS spectra were internally calibrated using porcine trypsin autolytic products (*m/z *842.509, *m/z *1045.564, *m/z *1940.935 and *m/z *2211.104) resulted in mass errors of less than 30 ppm. The MS peaks (MH^+^) were detected on minimum S/N ratio ≥ 20 and cluster area S/N threshold ≥ 25 without smoothing and raw spectrum filtering. Peptide precursor ions corresponding to contaminants including keratins and the trypsin autolytic products were excluded. The filtered precursor ions with a user-defined threshold (S/N ratio ≥ 50) were selected for the MS/MS scan. Fragmentation of precursor ions was performed using MS-MS 1 kV positive mode with CID on and argon/air as the collision gas. MS/MS spectra were accumulated from 3000 laser shots using default calibration with Glu-Fibrinopeptide B from 4700 Calibration Mixture (Applied Biosystems, USA). The MS/MS peaks were detected on minimum S/N ratio ≥ 3 and cluster area S/N threshold ≥ 15 with smoothing.

### Database Search

The MS and MS/MS data were loaded into the GPS Explorer™ software v3.5 (Applied Biosystems, Foster City, USA) and searched against NCBI database by Mascot search engine v1.9.05 (Matrix science, London, UK) using combined MS (peptide-mass-fingerprint approach) with MS/MS (*de novo *sequencing approach) analysis for protein identification. The following search parameters were used: monoisotopic peptide mass (MH^+^); 700–3500 Dalton; one missed cleavage per peptide; enzyme, trypsin/Lys-C; taxonomy, all taxonomy and green plants; p*I*, 0–14; precursor ion mass tolerance, 50 ppm; MS/MS fragment-ion mass tolerance, 0.1 Da; variable modifications, carbamidomethylation of cysteine, oxidation of methionine, acetylation of Lysine and arginine, mono-, di- and tri-methylation of Lysine were allowed. Known contaminant ions corresponding to trypsin and keratins were excluded from the peak lists before database searching. Top ten hits for each protein search were reported. For PTMs confirmation by MS/MS analysis, *De novo *Explorer™ software (Applied Biosystems, Foster City, USA) was used to deduce the amino acid sequence of selected peptide.

### Western blotting

Ten μg core histone mixtures were separated in SDS-PAGE gel, and transferred to a polyvinylidene difluoride (PVDF) membrane. The membranes were first blocked in 5% not-fat milk in TBS, and probed with specific primary antibody (1:1000). After three washes with TBST, the membranes were incubated with alkaline phosphatase-conjugated secondary antibody (goat-Anti-rabbit IgG-AP, Santa Cruz Biotechnology) at 1:2000 dilution in TBS. The signal is developed by the NBT/BCIP (Roche). Specific antibodies used in the experiments included: H3K18 acetylation (Upstate, 07–354), H3K23 acetylation (Upstate, 07–355), H3K4 trimethylation (MILLIPORE, 04–745), H3K27 trimethylation (LPBio, AR-0171), H3K36 trimethylation (Upstate, 05–801), H3K79 monomethylation (LPBio, AR-0172) and H3K79 dimethylation (LPBio, AR-0177), H4K8 acetylation (Upstate, 07–328), H4K12 acetylation (Upstate, 04–119) [45-47].

## Authors' contributions

WT and YT designed the study, carried out the experiment, conducted the analysis of histone modifications and drafted the manuscript. TS carried out mass spectrometry analysis. WC helped analyze the histone modifications. SS helped draft the manuscript. LH and NS conceived and designed the study and drafted the manuscript. All authors read and approved the final manuscript.
